# The effects of the herbal medicine Daikenchuto (TJ-100) after esophageal cancer resection, open-label, randomized controlled trial

**DOI:** 10.1007/s10388-017-0601-9

**Published:** 2017-12-20

**Authors:** Takeshi Nishino, Takahiro Yoshida, Masakazu Goto, Seiya Inoue, Takuya Minato, Satoshi Fujiwara, Yota Yamamoto, Yoshihito Furukita, Yasuhiro Yuasa, Hiromichi Yamai, Hirokazu Takechi, Hiroaki Toba, Hiromitsu Takizawa, Mitsuteru Yoshida, Junichi Seike, Takanori Miyoshi, Akira Tangoku

**Affiliations:** Department of Thoracic, Endocrine Surgery and Oncology, Institute of Biomedical Sciences, Tokushima University Graduate School, 3-18-15 Kuramoto-cho, Tokushima, 770-8503 Japan

**Keywords:** Esophageal cancer, Esophagectomy, Daikenchuto, TJ-100, Adrenomedullin

## Abstract

**Background:**

*Daikenchuto* (TJ-100), a traditional Japanese herbal medicine, is widely used in Japan. Its effects on gastrointestinal motility and microcirculation and its anti-inflammatory effect are known. The purpose of this prospective randomized controlled trial was to investigate the effect of TJ-100 after esophagectomy in esophageal cancer patients.

**Methods:**

Forty patients for whom subtotal esophageal resection for esophageal cancer was planned at our institute from March 2011 to August 2013 were enrolled and divided into two groups at the point of determination of the operation schedule after informed consent was obtained: a TJ-100 (15 g/day)-treated group (*n* = 20) and a control group (*n* = 20). The primary efficacy end-points were maintenance of the nutrition condition and the recovery of gastrointestinal function. The secondary efficacy end-points were the serum C-reactive protein (CRP) level and adrenomedullin level during the postoperative course, the incidence of postoperative complications, and the length of hospital stay after surgery.

**Results:**

We examined 39 patients because one patient in the TJ-100 group was judged as having unresectable cancer after surgery. The mean age of the TJ-100 group patients was significantly older than that of the control group patients.The rate of body weight decrease at postoperative day 21 was significantly suppressed in the TJ-100 group (3.6% vs. the control group: 7.0%, *p* = 0.014), but the serum albumin level was not significantly different between the groups. The recovery of gastrointestinal function regarding flatus, defecation, and oral intake showed no significant between-group differences, but postoperative bowel symptoms tended to be rare in the TJ-100 group. There was no significant between-group difference in the length of hospital stay after surgery. The serum CRP level at postoperative day 3 was 4.9 mg/dl in the TJ-100 group and 6.9 mg/dl in the control group, showing a tendency of a suppressed serum CRP level in the TJ-100 group (*p* = 0.126). The rate of increase in adrenomedullin tended to be high postoperatively, but there was no significant difference between the two groups.

**Conclusions:**

TJ-100 treatment after esophageal cancer resection has the effects of prompting the recovery of gastrointestinal motility and minimizing body weight loss, and it might suppress the excess inflammatory reaction related to surgery.

## Introduction

Esophageal resection for esophageal cancer is one of the most invasive surgical procedures for digestive cancer surgery, with a high incidence of morbidity and mortality. Improved surgical techniques and modern perioperative care have reduced the mortality rate, but there is still a high morbidity rate, which remains approx. 25–50% [1]. The prevention of postoperative complications is an important issue for the improvement of the prognoses of esophageal cancer patients [2].

The traditional Japanese herbal medicine *Daikenchuto* (TJ-100) has been used in Japan for the prevention and treatment of postoperative ileus [3]. Animal experiments have shown that the administration of TJ-100 accelerates gastrointestinal transit, increases intestinal blood flow, and has an anti-inflammatory effect [4–6]. Previous clinical studies have shown that perioperative TJ-100 administration helps prevent postoperative complications after colon cancer and gastric cancer surgery [7, 8]. Here we performed a randomized controlled study to determine the effects of TJ-100 after esophageal cancer surgery.

## Patients and methods

Forty patients for whom subtotal esophageal resection for esophageal cancer was planned at our institute from March 2011 to August 2013 were enrolled in this study. The patients were divided into two groups at the point of the determination of the operation schedule: after informed consent was obtained: TJ-100-treated group (the TJ-100 group, *n* = 20) and a control group (*n* = 20). The randomization was made using the envelope method without a blind technique. The patients in the control group were not administered any drugs or a placebo other than the surgical anesthesia. The inclusion criteria were as follows: confirmed esophageal carcinoma (the histology result did not matter), planned open subtotal esophageal resection and reconstruction by gastric tube or jejunum, age over 20 years (no upper limits), a performance status of 0 or 1, had maintained main internal organ (i.e. bone marrow, heart, liver, kidney, lung) function, no prior chemotherapy or radiotherapy for any malignancy, and had provided written informed consent. The exclusion criteria were as follows: planned laparoscopic surgery, emergent surgery, prior herbal medicine administration, inflammatory colitis (ulcerative colitis or Crohn’s disease), and pregnant or lactating women.

### Protocol

All patients were admitted to our hospital 5 days before surgery, and all received immune-nutrition therapy (the intake of a high-density liquid diet that contained omega-3 fatty acid) for 4 days. Conforming to the Japanese treatment guidelines for esophageal cancer, all patients were administered intravenous methylprednisolone just before the thoracotomy. The standard surgical procedure is open subtotal esophageal resection with a two- or three-area lymphadenectomy and reconstruction by gastric tube through the posterior mediastinum or a retro-sternal route.

All patients received a jejunostomy during the esophageal resection, and next day after surgery, they started receiving a semi-elemental diets (Racol^®^) from 10 ml/h (240 kcal/day) on postoperative day 1, and increased by 10 ml/h day-to-day until 60 ml/h (1440 kcal/day) on postoperative day 6. The TJ-100-treated patients were each injected with 15 g/day (5 g by 3 ×/day) of TJ-100 from the day after surgery until postoperative days 21. The control patients were not injected with TJ-100. The patients started oral intake on postoperative days 7 after per-oral contrast radiography, if there were no anastomosis problems and if no symptoms (bloating or nausea) were present. The meal was changed slightly every day until the patient could tolerate an ordinary diet. As the primary endpoints, we assessed the patients’ maintenance of nutrition condition including their body weight change and serum albumin levels. And more we assessed the recovery of patients’ gastrointestinal function including the number of days until the first flatus and first defecation from the end of surgery, and the days until becoming to be able to intake ordinary diet more than half of full diet (800 kcal) per a day. As secondary endpoints, we assessed the change in the patients’ serum C-reactive protein (CRP) level and plasma adrenomedullin (ADM) concentration during the postoperative course, the incidence of postoperative complications, and the length of hospital stay after surgery. Adverse events were defined according to the National Cancer Institute Common Terminology Criteria for Adverse Events (CTCAE, ver. 3.0).

The plasma ADM concentrations were measured by using an enzyme immunoassay kit (Phoenix Pharmaceuticals, Burlingame, CA) at four times points (pre-operation and postoperative days 1, 3, and 7). As the average plasma concentration of human ADM is not known, in order to identify the optimal dilution degree, we picked three random patients and measured the plasma ADM concentration of each at the above-mentioned four time points by one, three, and tenfolds, and we used the onefold dilution found in the near center of the standard curve (0.001–100 ng/ml), with all cases measured by onefold dilution.Duplicate measurements and averages of each data point were adopted. We compared the TJ-100 and control groups’ rate of increase in ADM, which was calculated as [postoperative value (ng/ml) − preoperative value (ng/ml)]/preoperative value (ng/ml).

### Statistical analysis

The sample size was calculated on the basis that the rate of body weight decreases at postoperative days 21 was expected to be 10% for the control group. In case the effect of reducing the decreasing rate of postoperative body weight is assumed to be 5% for the TJ-100 group with ± 5% standard deviation, the least number of patients to provide the 80% power necessary to confirm the superiority of a group was calculated to be 20 per group for a two-sided 1.25% significance level test.

The results are expressed as the median (interquartile range), and the statistical analyses were performed using Wilcoxon rank-sum test for quantitative parameters, and the Fisher’s exact probability test for qualitative parameters in Tables 1, 2 and 3. Statistical significance was set at *p* < 0.05.

**Table 1:** Patients characteristics and pre-operative data : The mean age of the TJ-100 group was significantly older than that of the control group (p=0.013). Regarding the gender, clinical stage, with/without preoperative therapy, and co-morbidity, there were no significant differences between the TJ-100 and control groups. And the preoperative data showed no significant between-group differences

	TJ-100 group (*n* = 19)	Control group (*n* = 20)	*p* value
Age (years old) Median (interquartile range)			
	68.0 (61.0–74.0)	60.5 (55.0–67.0)	0.018
Gender			
Male	17	16	0.661
Female	2	4	
Clinical stage			
I–II	6	8	0.741
III–IV	13	12	
Neo-adjuvant chemotherapy			
+	15	15	0.535
−	4	5	
Co-mobidities	13	11	0.298
Cardiovascular disease	3	1	0.283
Respiratory disease	1	1	0.744
Diabetes mellitus	2	2	0.678
Liver disease	5	4	0.465
Renal disease	0	2	0.256
Neuromuscular disease	3	1	0.283
Other disease	2	3	0.525
Pre-operative data Median (interquartile range)			
Body weight (kg)	62.5 (55.9–70.5)	60.2 (50.3–69.6)	0.588
Body mass index	24.0 (19.5–24.8)	22.1 (19.9–24.2)	0.283
Albumin (g/dl)	3.6 (3.5–3.9)	3.5 (3.3–3.9)	0.324
CRP (mg/dl)	0.10 (0.07–0.15)	0.06 (0.05–0.12)	0.123

**Table 2:** Operation data: There were no significant differences in the surgical findings, postoperative complications, but the case who developed postoperative bowel symptom and elongation of hospital stay due to abdominal symptoms tended to be frequent in control group without significant differences

	TJ-100 group (*n* = 19)	Control group (*n* = 20)	*p* value
Reconstruction route			
Anterior sternal	9	9	0.247
Posterior mediastinum	10	11	
Intraoperative bleeding (ml) [median (interquartile range)]	160.0 (100.0–270.0)	209.5 (140.0–366.0)	0.222
Operation time (min) [median (interquartile range)]	366.0 (295.0–409.0)	348.5 (306.2–419.8)	0.983
Postoperative complications	10	7	0.341
Anastomosis leakage	1	0	0.487
Surgical site infection	0	3	0.231
Pneumonia	2	0	0.231
Anastomosis stenosis	0	2	0.487
Recurrent nerve paralysis	2	1	0.605
Ileus	0	1	0.513
Other	5	3	0.451
Developed bowel symptoms	1	5	0.076
Elongation of hospital stay due to postoperative bowel symptoms	0	4	0.056

**Table 3:** The recovery of gastrointestinal function: Regarding the recovery of gastrointestinal function, the number of days until first flatus and first defecation, there was no significant differences, there was no significant differences. And also the number of days until the patient was able to tolerate an ordinary diet was same between both groups

	TJ-100 group (*n* = 19)	Control group (*n* = 20)	*p* value
Days until first flatus (days)	3.0 (2.0–4.0)	3.0 (2.0–4.0)	0.487
Days until first defecation (days)	6.0 (4.0–8.0)	4.5 (3.3–6.0)	0.108
Days until intake ordinary diet (days)	14.0 (13.0–16.0)	14.5 (13.0–20.0)	0.773
Hospital stay after surgery (days)	23.0 (21.0–35.0)	21.0 (18.3–28.3)	0.189

Median (interquartile range)

The rate of body weight decreases are compared by medians at four times points (postoperative day 3, 7, 14, and 21) using Wilcoxon rank-sum test with the Bonferroni correction considering multiplicity for each comparison at a significant level of *α*/*m* (*α* was set at 0.05 and *m* was set to 4), accordingly statistical significance was set at *p* < 0.0125. All statistical analysis was performed using SPSS software (IBM, Armonk, NY).

## Results

Since one patient in the TJ-100 group was judged as having unresectable cancer after surgery, we examined 19 patients in the TJ-100 group and 20 patients in the control group. The characteristics of all patients are summarized in Table 1. The median age of the TJ-100 group (68.0 years old) was significantly older than that of the control group (60.5 years old) (*p* = 0.018). Regarding the gender, clinical stage, with/without preoperative therapy, and co-morbidity, there were no significant differences between the TJ-100 and control groups.

Regarding the preoperative data, the patients’ body mass index, body weight, albumin level and serum CRP values showed no significant between-group differences (Table 1).There were also no significant differences in the surgical findings (reconstruction route, blood loss, operating time). Regarding the rate of postoperative complications (e.g., anastomosis leakage, surgical site infection, pneumonia, and anastomosis stenosis), there were no significant between group differences (Table 2).

**Fig. 1:**
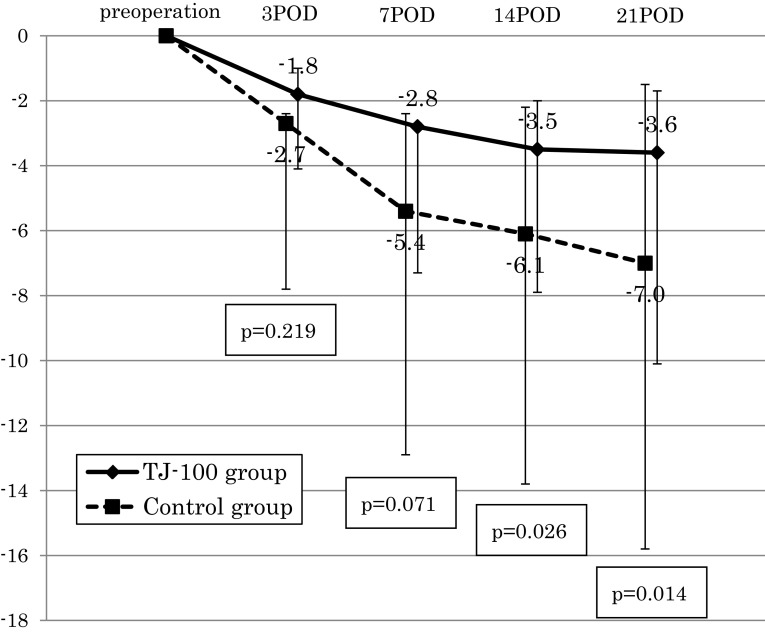
The perioperative changes in the rate of body weight decreases The differences of perioperative changes in the rate of body weight decreases between the TJ-100 and control groups appeared gradually from postoperative days 3 and at postoperative days 21, the control patients showed significantly more decreased body weight compared to the TJ-100 group (*p* = 0.014)

Figure 1 illustrates the perioperative changes in the rate of body weight decreases. Differences between the TJ-100 and control groups appeared gradually from postoperative days 3. At postoperative day 21, the control patients showed significantly more decreased body weight compared to the TJ-100 group (*p* = 0.014); the rate of body weight decreases of the TJ-100 group was 3.6%, whereas that of the control group was 7.0% at postoperative day 21. Thus, the administration of TJ-100 significantly reduced the loss of body weight after esophagectomy.

**Fig. 2:**
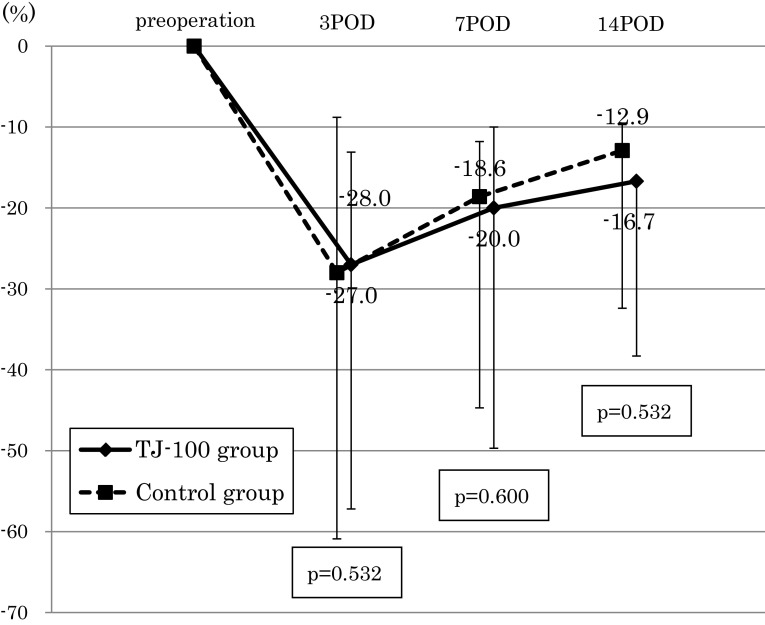
The perioperative changes in the rate of the serum albumin decreases The perioperative change in the rate of the serum albumin decreases was not significantly different between the two groups

**Fig. 3:**
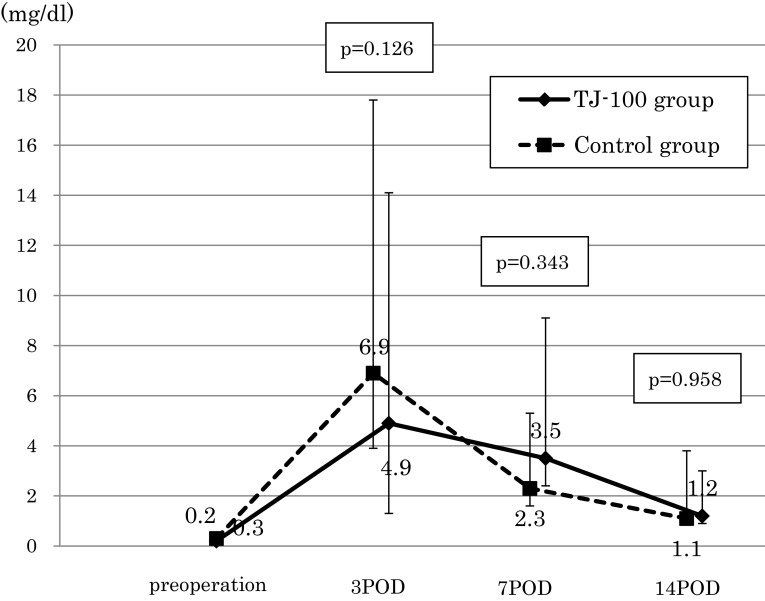
The perioperative changes in the serum CRP levels At postoperative day 3, it showed a tendency for a suppressed serum CRP level in the TJ-100 group compared to the control group (*p* = 0.126)

**Fig. 4:**
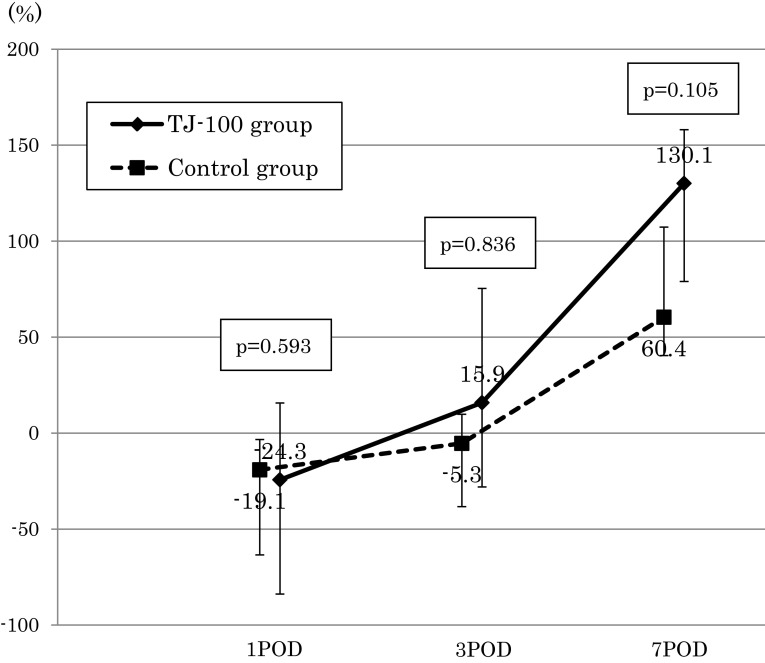
The perioperative changes in the rate of serum ADM concentration increases The rate of increase in the ADM concentration tended to be high postoperatively, it showed no significant difference between the two patient groups

The perioperative changes in the rate of the serum albumin decreases were not significantly different between the two groups (Fig. 2). We assessed the serum CRP level with the exclusion of six patients in whom an inflammatory complication occurred (anastomosis leakage, surgical site infection, and pneumonia). At postoperative day 3, the serum CRP level of the TJ-100 group was 4.9 mg/dl and that of the control group was 6.9 mg/dl, thus showing a tendency for a suppressed serum CRP level in the TJ-100 group compared to the control group (*p* = 0.126) (Fig. 3). The perioperative changes in the rate of serum ADM concentration increases tended to be high in the TJ-100 group postoperatively, it showed no significant difference between the two patient groups (Fig. 4).

Regarding the recovery of gastrointestinal function, the median number of days until first flatus was 3.0 (2.0–4.0) days in the TJ-100 group and 3.0 (2.0–4.0) days in the control group, a nonsignificant difference (n.s.).The number of days until first defecation was 6.0 (4.0–8.0) days in the TJ-100 group and 4.5 (3.3–6.0) days in the control group (n.s.). The number of days until the patient was able to tolerate an ordinary diet was 18.1 (± 11.9) days in the TJ-100 group and 18.3 (± 11.5) days in the control group (n.s.). A postoperative bowel symptom was observed in only one patient in the TJ-100 group and in five patients in the control group (n.s., *p* = 0.076). Postoperative ileus developed in no patients in the TJ-100 group and one in the control group. Elongation of hospital stay due to abdominal symptoms was observed in no patients in the TJ-100 group and four in the control group (n.s., *p* = 0.056).

There was no significant difference in the median length of hospital stay after surgery between the TJ-100 group and the control group: 23.0 (21.0–35.0) days and 21.0 (18.3–28.3) days, respectively. The patients for whom discharge was delayed due to abdominal symptoms (e.g., abdominal distention, constipation, abdominal discomfort) were four control patients and none of the TJ-100-administered patients. The TJ-100 treatment tended to improve postoperative abdominal symptoms, but there was no significant between-group difference in the length of postoperative hospital stay, as noted above (Table 3).

## Discussion

The herbal medicine *Daikenchuto* (TJ-100) contains *kankyo* (dried ginger), *sansho* (Japanese pepper), *ninjin* (ginseng), and *koui* (Saccharum granorum). It has been used widely for the prevention and treatment of adhesive and paralytic ileus and constipation in Japan. The biological mechanism underlying the effects of TJ-100 has been investigated in animal studies; in fact, among the traditional herbal medicines, TJ-100 has been investigated the most extensively. Its main effects are the acceleration of gastrointestinal transit, an increase in intestinal blood flow, and an anti-inflammation effect.

The acceleration of gastrointestinal transit by TJ-100 occurs via the stimulation of muscarine receptors by acetylcholine hormone, promoted by the binding of serotonin to 5-hydroxytryptamine three receptor and four receptor [7], the stimulation of the secretion of motilin from motilin secretion cells (Mo cells) in the intestinal mucosa [8], and the isolation of substance P through the transient receptor potential cation channel subfamily V member (TRPV1) channels distributed in the sensory nerves of the intestinal lumen [9]. Increased intestinal blood flow is brought about by the production of ADM and calcitonin gene-related peptide (CGRP) [10, 11]. The anti-inflammation effect of TJ-100 is exhibited through ADM as an inhibitor of inflammatory cytokines, interferon-γ, and tumor necrosis factor (TNF)-α. The release of ADM plays an important role in the beneficial effect of TJ-100 [12].

Several clinical trials of the effectiveness of TJ-100 treatment have recently been performed, and the evidence of this medicine’s benefits has gradually accumulated. Regarding the effect of the acceleration of gastrointestinal transit by TJ-100 administration, Ito et al. [6] performed a randomized controlled trial to investigate the prevention of postoperative ileus by a 14-day regimen of TJ-100. They reported a significantly lower rate of re-operation for postoperative ileus in the TJ-100-treated patients compared to the placebo group. Endo et al. [13] evaluated the effect of TJ-100 treatment on the emptying and motility ability of jejunal pouch interposition after total gastrectomy. They observed that the TJ-100 treatment significantly improved the rate of stasis symptoms and the emptying and motility ability of the jejunal pouch. Manabe et al. [14] examined the effect of TJ-100 on gastrointestinal and colon transit and bowel function as validated by scintigraphy in healthy humans. They reported that TJ-100 significantly accelerated colonic filling and ascending colon emptying.

Regarding the anti-inflammatory effect of TJ-100, Yoshikawa et al. [3] reported that the postoperative administration of TJ-100 after surgery for colorectal cancer significantly suppressed the CRP level and shortened the time until first flatus after surgery. Nishi et al. [15] observed that TJ-100 treatment after liver resection significantly suppressed the CRP level was brought about by the suppression of inflammatory cytokines by ADM.

The peptide ADM is a member of the calcitonin family,and it is comprised of 53 amino acids. It was initially isolated from a human pheochromocytoma. ADM is distributed in the vascular endothelium and gastrointestinal tract, and it plays an important role in the regulation of microcirculation [16]. ADM has various bioactivation functions, including a hypotensive effect by vasodilation and roles in cell migration, differential regulation, anti-inflammation, and increasing vascular flow. Research using rodents revealed that ADM is induced by TJ-100 administration, and that TJ-100 has many effects, e.g., accelerating gastrointestinal transit and increasing intestinal blood flow, and an anti-inflammation effect [17]. The concentration of ADM in human blood plasma has not been established. The results obtained in our present patient series do not confirm a significant increase in the plasma concentration of ADM after TJ-100 treatment, in light of the large variance of ADM concentrations observed after surgery.

Our investigation did show a significant prevention of body weight loss after esophagectomy by TJ-100 treatment. This effect was presumably brought about by the rapid improvement of gastrointestinal function and the promotion of the absorption of enteral nutrition after surgery, but we did not observe a significant difference in the improvement of the albumin level as a nutrition indicator, and thus the effect of TJ-100 on nutritional improvement remains unknown. It is desirable to examine other indicators that have a more rapid turn-over; e.g., pre-albumin, transferrin, and retinol acid binding protein.

We found that the peak serum CRP level after surgery tended to be suppressed by the TJ-100 treatment. This effect was presumably due to the inhibition of inflammatory cytokines by ADM induced by TJ-100 treatment, but there was no significant difference in the rate of increase of ADM between the control and TJ-100 groups. Our findings thus did not reveal the mechanism of the anti-inflammatory effect of TJ-100. Further large-scale investigations should be performed to reveal this anti-inflammatory mechanism.

## Conclusion

TJ-100 treatment after esophageal cancer resection promoted the recovery of gastrointestinal motility and minimized body weight loss, and it might suppress the excessive inflammatory reaction related to surgery.
